# DevR (DosR) mimetic peptides impair transcriptional regulation and survival of *Mycobacterium tuberculosis* under hypoxia by inhibiting the autokinase activity of DevS sensor kinase

**DOI:** 10.1186/1471-2180-14-195

**Published:** 2014-07-21

**Authors:** Kohinoor Kaur, Neetu Kumra Taneja, Sakshi Dhingra, Jaya S Tyagi

**Affiliations:** 1 Department of Biotechnology, All India Institute of Medical Sciences, New Delhi, India; 2 Present address: National Institute of Food Technology Entrepreneurship and Management (Ministry of Food Processing Industries, Government of India) Plot No. 97, Sector-56, HSIIDC Industrial Estate, Kundli, District Sonepat, Sonepat, Haryana 131028, India

**Keywords:** Phage display, DevRS peptides, Inhibition of autokinase, Hypoxia

## Abstract

**Background:**

Two-component systems have emerged as compelling targets for antibacterial drug design for a number of reasons including the distinct histidine phosphorylation property of their constituent sensor kinases. The DevR-DevS/DosT two component system of *Mycobacterium tuberculosis (M. tb)* is essential for survival under hypoxia, a stress associated with dormancy development *in vivo*. In the present study a combinatorial peptide phage display library was screened for DevS histidine kinase interacting peptides with the aim of isolating inhibitors of DevR-DevS signaling.

**Results:**

DevS binding peptides were identified from a phage display library after three rounds of panning using DevS as bait. The peptides showed sequence similarity with conserved residues in the N-terminal domain of DevR and suggested that they may represent interacting surfaces between DevS and DevR. Two DevR mimetic peptides were found to specifically inhibit DevR-dependent transcriptional activity and restrict the hypoxic survival of *M. tb.* The mechanism of peptide action is majorly attributed to an inhibition of DevS autokinase activity.

**Conclusions:**

These findings demonstrate that DevR mimetic peptides impede DevS activation and that intercepting DevS activation at an early step in the signaling cascade impairs *M. tb* survival in a hypoxia persistence model*.*

## Background

The DevR dormancy regulon of *Mycobacterium tuberculosis* (*M. tb*) is a transcriptional program induced by low oxygen tension, nitric oxide (NO), carbon monoxide (CO) or vitamin C treatment that enables bacterial adaptation and survival during periods of non-replicating persistence in *in vitro* models of dormancy [1-4]. This regulon is believed to assist bacterial survival during latent tuberculosis (TB) infection, a chronic asymptomatic state that afflicts one-third of the global population [5]. The expression of the ~48 gene regulon is coordinated by DevR (also known as DosR) which binds to target sites on DNA and induces transcription after its phosphorylation by DevS and/or DosT [3,6-8]. DevS and DosT sensor kinases were therefore proposed as targets for the development of novel antibacterial compounds against dormant bacteria [9]. DevS was also predicted to be a potential persistence target inhibiting dormant *M. tb* using an in silico approach [10].

Two component systems are considered as compelling targets for drug design due to a number of reasons including their absence in higher eukaryotes, the difference in bacterial two-component signaling as compared to signaling pathways in eukaryotes, and most importantly, the essential roles they play in bacterial viability, virulence and drug resistance. Inhibitors of bacterial histidine kinases have been reported in the literature [11-13] but most suffer from the drawback of being extremely hydrophobic. Most inhibitors exhibit multiple mechanisms of action including surfactant and membrane damaging properties that are independent of two-component system inhibition. The high hydrophobicity of these molecules makes formulation and delivery of the compounds extremely difficult. Furthermore, the compounds showed excessive plasma protein binding and minimal bioavailability and were ineffective in standard *in vivo* infection models. In contrast, peptides are believed to confer several advantages, such as high target specificity, lower accumulation in tissues and lower toxicity coupled with new efficient synthesis strategies and low monomer prices. A recent study identified potential PhoQ inhibitor compounds that inhibited autophosphorylation and also inhibited severe keratoconjunctival inflammation in mice inoculated with *Shigella flexneri*[14], suggesting that two-component systems are potential targets for the development of drugs against bacteria.

We had earlier described the properties of a DevR binding peptide DevRS1 identified through phage display technology that inhibited DevR function [15]. Here we report the identification of peptides that majorly target the autokinase function of DevS sensor and inhibit DevR-mediated transcription and survival of *M. tb* under hypoxia*.* The ability to interfere with DevR-DevS function at more than one step demonstrates that this two-component system is a rich target for developing inhibitor(s) to effectively block *M. tb* adaptation to hypoxia, a potent dormancy signal.

## Results and discussion

### Isolation of DevS binding peptides from a phage display peptide library

**Figure 1:**
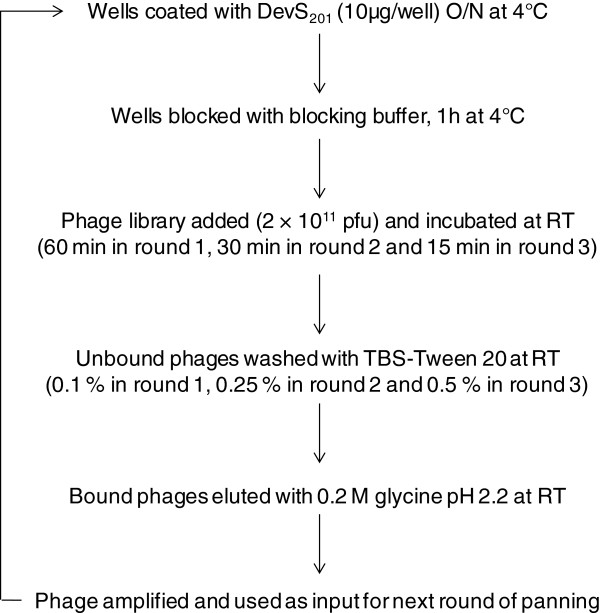
**Scheme for isolating DevS binding phages.** Two wells of a 96 well plate were coated with DevS_201_ protein and incubated overnight at 4°C. Next, the wells were blocked for 1 hr at 4°C. Phage library containing 2 × 10^11^ pfu were added to the wells and incubated at room temperature. The unbound phages were removed by washing with TBS-Tween 20. The bound phages were eluted with 0.2 M glycine pH 2.2. The eluted phages were amplified and used as input for the next round of panning. The panning conditions were made stringent in each successive round of panning by decreasing the time of incubation of phage library with the protein and by increasing the concentration of Tween-20 in the washing steps.

**Figure 2:**
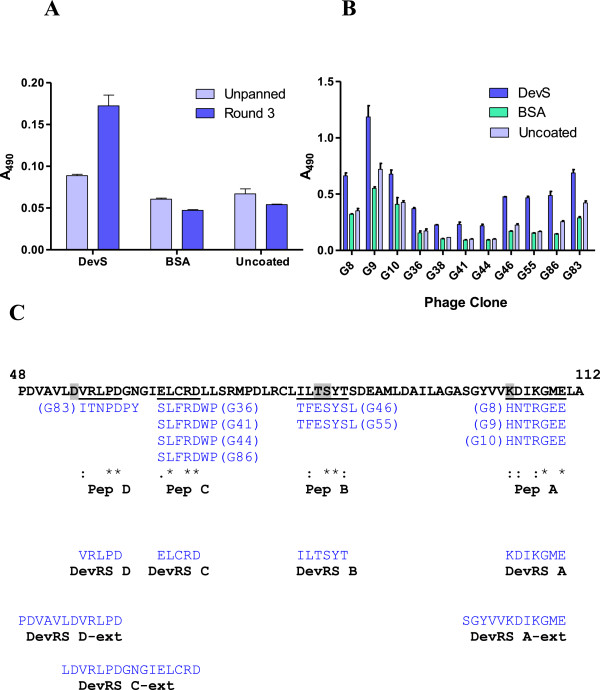
**Identification of DevS binding peptides using biopanning. (A)** Enrichment of phages binding to DevS protein. Equal numbers of phage particles (10^11^) before (unpanned library) and after three rounds of biopanning on DevS (Pan 3) were incubated in wells coated with DevS or BSA or left uncoated. The unbound phages were removed by washing the wells with TBS containing 0.5% Tween 20 and the bound phages were detected with HRP-conjugated anti-M13 antibody (Amersham) and o-Phenylenediamine substrate. The assays were performed in triplicate and the mean ± SD values are plotted. **(B)** Phage ELISA of selected phage clones. Phage clones displaying DevS-binding peptides were used in ELISA. Equal numbers of phage particles after amplification were incubated in wells coated with DevS or BSA or left uncoated. The bound phages were detected as described above. **(C)** Alignment of peptides identified by DevS panning with the sequence of DevR. Functionally important and conserved residues: D54, phosphorylation site; T82, K104, residues involved in phosphorylation induced conformational changes, are highlighted in grey. Screening a phage display library for DevS binders identified the 'Pep’ peptides. DevRS peptides were designed on the basis of DevR sequence. CLUSTALW-based alignment of the 'DevRS’ peptides with the DevR sequence is shown. *, positions having a fully conserved residue; **:**, conservation between groups of strongly similar properties; **.**, conservation between groups of weakly similar properties. DevRS B-ext could not be analyzed due to the inability to prepare soluble peptide.

The cytoplasmic domain of DevS (named as DevS_201_), that transfers the activating phosphosignal to DevR [7], was used to screen a phage display peptide library using a panning strategy described in Figure 1. A total of 110 phages from the glycine eluate of DevS_201_ plate from the third round of panning were individually amplified and screened by ELISA to assess their DevS binding specificity. DevS binder phages were enriched relative to the unpanned library after three rounds of panning (Figure 2A). Thirty clones (G8, G9 etc.) showed 2–4 fold higher binding to DevS_201_ versus BSA or uncoated wells (Figure 2B). DNA sequence analysis of the peptide coding sequences in these phages revealed four peptide sequences, namely, HNTRGEE (Pep A), TFESYSL (Pep B), SLFRDWP (Pep C) and ITNPDPY (Pep D) that were repeated 2–4 times (Figure 2C).

### Activity of 'Pep’ peptides

The 'Pep’ peptides identified by biopanning were tested for activity in DevS autokinase assays. Pep A, B and D peptides were found to inhibit DevS autokinase activity to varying degrees (see Additional file 1). However, the data showed a great variation and was not reproducible among the assay replicates for individual peptides. This could be attributed to protein aggregation that was observed upon addition of peptides to the assays. However, Pep C did not inhibit autokinase activity at any of the concentrations tested.

### Similarity between PepA, PepB, PepC and PepD sequences and DevR catalytic centre

The 'Pep’ peptides identified by panning were aligned next with the protein sequence of DevR. Three peptides appeared to align near the Asp-54, Thr-82 and Lys-104 residues located in the N-terminal domain of DevR (Figure 2C). Asp-54 is the site of phosphorylation and Thr-82 and Lys-104 residues are involved in phosphorylation-induced conformational changes [7,16]. The partial identity noted between 'Pep’ peptides and DevR protein sequences suggested that these peptides might represent surfaces on the N-terminal domain of DevR (DevR_N_) that could interact with DevS.

### The DevR_N_ domain interacts with the cytosolic domain of DevS

**Figure 3:**
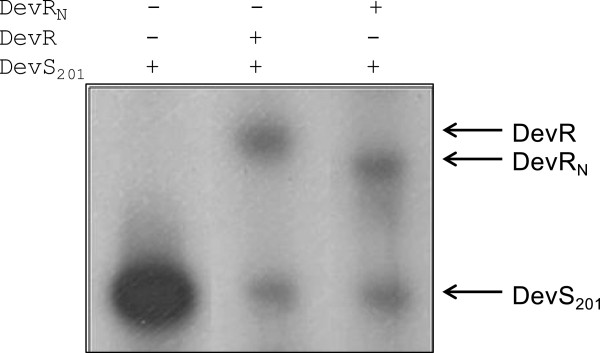
**In vitro phosphotransfer assay.** Reaction mixtures containing purified DevS ~ ^32^P and GST- DevR full length/GST - DevR_N_ proteins were incubated at 25°C for 2 mins. Samples were analysed by 15% SDS-PAGE and autoradiography.

Because DevR_N_, in particular its Asp54 residue, is involved in interaction of DevR and DevS proteins of *M. smegmatis*[17], we investigated the possibility of a similar interaction occurring between DevR_N_ and DevS proteins of *M. tb*. An in vitro phosphotransfer assay was performed using DevR_N_ and the cytosolic domain of DevS protein. A rapid and comparable transfer of the phosphosignal was noted from *M. tb* DevS to either DevR or DevR_N_, which established that the N terminal domain of DevR interacts with DevS (Figure 3).

Having demonstrated that (1) 'Pep’ peptides exhibit a modest anti-DevR activity (Additional file 1), (2) 'Pep’ peptides bear sequence similarity with conserved sequences in the N-terminal domain of DevR (Figure 2C) and (3) DevR_N_ interacts with DevS (Figure 3), we proceeded to synthesize 'DevR mimetic’ peptides that mapped in the same regions as the 'Pep’ peptides and were of identical sequence to DevR. The assumption was that DevR 'mimetic’ peptides would interact with greater affinity with DevS as compared to the 'Pep’ peptide sequences. The synthesized peptides were named as DevRS A (KDIKGME), DevRS B (ILTSYT), DevRS C (ELCRD) and DevRS D (VRLPD). Extended 10–16 mer versions of the DevRS peptides were also synthesized (peptides DevRS A-ext, C-ext, D-ext) assuming that they may afford better binding characteristics (Figure 2C). Due to the problem of protein aggregation observed in assays containing the 'Pep’ series of peptides, only extended 'DevRS’ and 'DevRS-ext’ peptides were characterized further. Moreover, these extended peptides were likely to afford better binding characteristics if indeed they represented interacting regions.

### 'DevRS’ peptides inhibit DevR-mediated transcription

**Figure 4:**
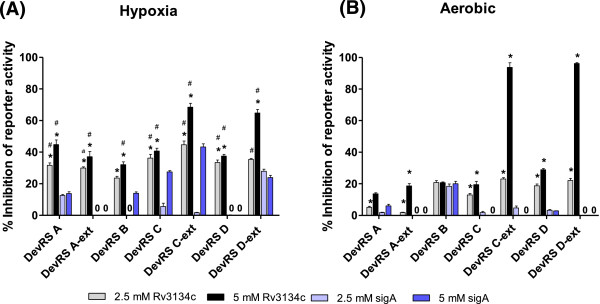
**Peptides inhibit DevR-regulated promoter activity.** Effect of peptides on *Rv3134c* and *sigA* promoter activity in *M.tb* cultures was assessed under hypoxic **(A)** and aerobic **(B)** conditions. The data represents the mean ± SD of two independent experiments. * denotes a significant difference (p < 0.05) in the activity of *Rv3134c* and *sigA* promoters under hypoxic and aerobic conditions based on a Students’ *t*-test. For example, DevRS A peptide-mediated inhibition (at 5 mM concentration) of the *Rv3134c* promoter under hypoxic and aerobic conditions is ~ 45% and 14%, which is significantly higher than ~ 14% and 6% inhibition of *sigA* promoter activity under identical conditions. # denotes a significant difference (p <0.05) in *Rv3134c* promoter activity under hypoxic and aerobic conditions based on a Student’s *t*-test. For example, DevRS A peptide-mediated inhibition (at 2.5 mM and 5 mM concentration) under hypoxia is 30% and 45%, which is significantly higher than 5% & 15% inhibition observed under aerobic conditions. '0’ values on top of some bars indicate no detectable inhibition. Non-specific inhibition of transcription (measured using *sigA* promoter reporter plasmid) was not observed for two peptides, DevRS A-ext and DevRS D peptides.

DevRS A, B, C and D and DevRS A-ext, C-ext and D-ext peptides were assessed for their ability to inhibit DevR-regulated transcription using a *M. tb* GFP reporter assay. The reporter assay measures the activity of the *Rv3134c* promoter, a well characterized DevR-dependent promoter [18]. Two of the peptides that were tested, namely DevRS A-ext and DevRS D, significantly (p < 0.05) inhibited *Rv3134c* promoter activity up to ~40% under hypoxic conditions and ~20-30% inhibition under aerobic conditions (Figure 4A and B). Both these peptides map at or near the conserved sequences of DevR that are involved in phosphorylation and are believed to mediate a conformational change during its activation. The observed inhibition of promoter activity in aerobic cultures (~20-30%, Figure 4B) is ascribed to hypoxia development in standing cultures reported earlier and not to aerobic inhibition per se [18,19]. It was noteworthy that both DevRS A-ext and DevRS D peptides did not inhibit *sigA* promoter activity (not regulated by DevR) under identical conditions, establishing the target specificity of these peptides for DevR. However, the remaining peptides inhibited the *Rv3134c* promoter (up to ~60% under hypoxia, Figure 4A), but caused non-specific inhibition of the *sigA* promoter and/or were inhibitory under aerobic conditions (up to ~90%), suggesting a lack of target specificity (Figure 4A and B). The specific inhibition of the *Rv3134c* promoter by DevRS A-ext and DevRS D peptides (Figure 4) implies that the expression of other members of the DevR regulon would also be impaired [19,20] and accordingly these two peptides were characterized further in viability assays.

### 'DevRS’ peptides specifically inhibit hypoxic survival of *M. tuberculosis*

**Figure 5:**
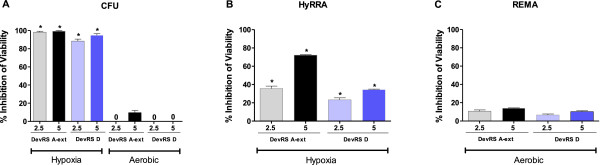
**Peptides inhibit hypoxic survival of *M. tb*.** Effect of peptides on viability of *M. tuberculosis* under hypoxic and aerobic conditions as determined by CFU **(A)**, Resazurin reduction assays HyRRA – hypoxic conditions **(B)** and REMA- aerobic conditions **(C)**. The data represents the mean and standard deviation of two independent experiments. '0’ on top of some of the bars indicates lack of detectable inhibition. *denotes p value of <0.05 for the difference in peptide-mediated inhibition of viability under hypoxia vs. aerobic conditions by CFU and/Resazurin reduction assays based on a Student’s *t*-test.

DevRS signaling is established to be essential for *M. tb* viability during hypoxia and not under aerobic conditions [21,22]. Therefore, DevRS A-ext and DevRS D peptides were evaluated for anti –TB activity using CFU assays and Resazurin reduction-based assays (REMA and HyRRA) as described in the Methods section. The REMA and HyRRA assays are very useful for screening anti-tubercular anti-dormancy compounds under aerobic and hypoxic conditions respectively and can be safely performed in high-throughput format in *M. smegmatis*, *M. bovis BCG* and *M. tb*[23]. In the CFU assay, DevRS A-ext and DevRS D peptides severely compromised hypoxic survival *M. tb* (~85 to 95% inhibition with respect to the DMSO control) in contrast to only a modest reduction in CFU yields in aerobic cultures (none to ~10% inhibition), thereby confirming target specificity (Figure 5A). The Resazurin reduction assays confirmed the target specificity of both the peptides; ~40 to 70% loss in viability was observed in the presence of each of these peptides in the hypoxic HYRRA assay as compared to a modest inhibition in the aerobic REMA assay (~15% to no inhibition, Figure 5B and C). These findings are consistent with our earlier observation that the HyRRA assay truly reports the viability of drug/inhibitor-treated dormant cultures [23]. A greater loss in peptide-mediated bacterial viability was observed in the CFU assay and it might be explained by the fact that inhibitor-treated bacteria were unable to grow on solid medium plates in the CFU assay but were maintained in viable form in broth cultures in the HyRRA/REMA assays as discussed earlier [23].

### 'DevRS’ peptides inhibit DevS autokinase activity

Auto phosphorylation of the sensor kinase is the first step in the DevS/DosT-DevR signaling cascade. To decipher the mechanism of action of the peptides, DevRS A-ext and DevRS D were tested in an *in vitro* ATP-dependent autokinase assay as described [7]. The addition of DevRS A-ext and DevRS D in the reaction inhibited DevS autokinase activity by 55% and 37% respectively (see Additional file 2). Our findings suggest that the inhibition of autokinase activity by DevRS A-ext and DevRS D is caused by peptide binding at/near the DevS phosphorylation site.

In addition to inhibiting autokinase activity (above), the peptides could in principle also inhibit transfer of the phosphosignal from DevS ~ P to DevR. However, neither DevRS A-ext nor DevRS D inhibited this reaction in an *in vitro* phosphotransfer assay (data not shown). Based on these findings, the observed defects in hypoxic adaptation of *M. tb* cultures are majorly attributed to a peptide-mediated block in the autokinase activity of DevS sensor kinase. Because (1) of the high sequence homology between DevS and DosT kinases, (2) DosT, in addition to DevS, can activate DevR through its autokinase activity under hypoxia [3,6,8,24] and (3) exposure to DevRS A-ext and DevRS D peptides results in a severe loss of hypoxic viability (Figure 5), we further propose that these two peptides inhibit the activities of both DevS and DosT kinases.

### Cytotoxicity of 'DevRS’ peptides

**Figure 6:**
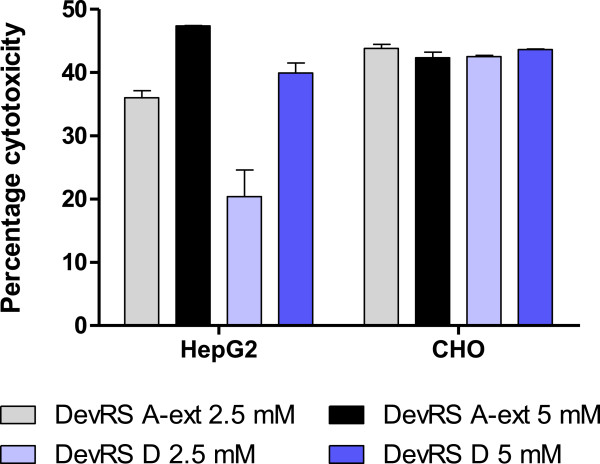
**Cytotoxicity of peptides.** Percentage cytotoxicity due to exposure to peptides was assessed in HepG2 and CHO cell lines using the MTT assay. The cells were exposed to DevRS A-ext and DevRS D peptides at 2.5 mM and 5 mM concentration in 96-well plate format for 48 h. Percentage cytotoxicity is shown with respect to the DMSO control as mean ± SD for duplicate values.

DevRS A-ext and DevRS D peptides were assessed next for their cytotoxicity in CHO and HepG2 mammalian cell lines using the MTT assay as described earlier [15]. Both the peptides were cytotoxic in the range of 20-40% at 5 mM and 2.5 mM concentration relative to the DMSO vehicle control (Figure 6).

## Conclusions

**Figure 7:**
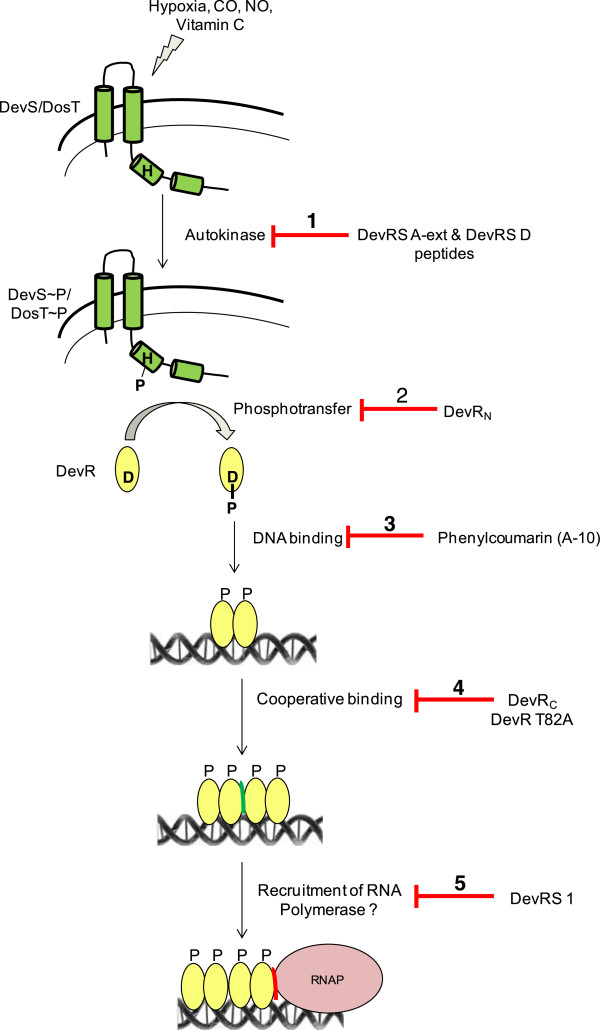
**Interception of the DevRS signaling cascade.** Hypoxia, NO, CO, and Vitamin C are sensed by DevS and DosT kinases (represented as membrane-associated sensors), resulting in the transfer of the phosphosignal from the sensors to DevR leading to its activation. Phosphorylated DevR binds to target gene promoters and elicits regulon activation. The red line/bars indicate the various steps in this signalling cascade whose interception results in a defect in the hypoxic adaptation of *M. tb*. ***Step 1***, the inhibition of DevS/DosT autokinase activity by DevRS A-ext and DevRS D peptides (this study) interrupts the initial step in two-component signalling. ***Step 2***, DevR_N_ protein competes with full-length DevR protein at the phosphotransfer step to inhibit bacterial adaptation [21]. ***Step 3***, phenylcoumarin A-10 functions by inhibiting the binding of DevR to DNA [20]. ***Step 4***, the cooperative interaction of DevR with DNA (green interface between 2 dimers of DevR) is required for robust induction [16,25], and serves as a novel inhibition step. ***Step 5***, a DevR interacting peptide DevRS1 [15] likely exerts its inhibitory effect by blocking the between DevR - RNA polymerase interaction (red interface).

In conclusion, the observed peptide-mediated inhibition, albeit at millimolar concentrations, demonstrates that interference of DevS/DosT signaling at the step of autokinase activity severely attenuates *M. tb* adaptation and survival under hypoxia, a condition that prevails within granulomas and is a likely trigger for bacterial dormancy initiation and maintenance *in vivo* during latent TB. We have earlier demonstrated that phenylcoumarin- or peptide-mediated blocking of DevR function severely impairs *M. tb* adaptation to hypoxia [15,20]. The small molecule inhibited DevR function by preventing its binding to specific motifs on DNA while the mode of action of the DevR binding peptide is yet unknown. In another approach, the isolated N-terminal domain of DevR functioned as a novel competitive inhibitor that interfered with signal transfer from DevS to native DevR. Competitive signaling resulted in a defect in DevR mediated transcriptional response and a loss of hypoxic viability [21]. In the present study, we show that inhibition of DevS autokinase activity at an early step of the signaling cascade interferes with DevRS function and significantly reduces the hypoxic viability of bacteria. Thus the DevR-DevS system is a rich target to develop molecules that interfere with one of many steps in the signaling cascade during *M. tb* adaptation to hypoxia (Figure 7)*.* Our findings further suggest that phage display technology is a powerful approach for designing inhibitors and is likely to be useful for inhibiting the activity of other bacterial two-component systems.

## Methods

### Screening of phage display library using cytoplasmic domain of DevS as bait

The recombinant cytosolic portion of DevS protein DevS_201_ (amino acids 377 to 578), was overexpressed and purified from *E. coli* BL21 harboring recombinant plasmid pSCS201 as described earlier [7]. The Ph.D.-7 Phage display peptide library kit (New England Biolabs Inc., Beverely, MA, USA) was screened by biopanning using the manufacturer’s instructions with a few modifications as outlined in Figure 1. Briefly, three rounds of panning were performed on a polystyrene 96-well micro titer plate wherein duplicate wells were coated with purified protein DevS_201_ -His_6_ (10 μg/well) and the phage library (2 × 10^11^ phages) was incubated with the immobilized protein to allow binding of the phage particles. Decreasing the time of incubation with DevS_201_ protein and increasing the percentage of Tween-20 in the washing buffer in each successive round of panning achieved the stringent selection of high affinity binder phages. Three rounds of panning were performed in all and each time the phages in the glycine elution (0.2 M glycine, pH 2.2) were amplified and used as an input for the next round of panning. The titer of phages in the various elutions was determined according to the manufacturer’s instructions and the input phage number was maintained during each round of panning.

### ELISA

The high affinity binder phages were identified using ELISA. Briefly, individual phage plaques were picked from the glycine elution of the last round of panning, amplified and the culture supernatants (containing phages) were screened for binding to DevS_201_ or BSA or to plastic. Briefly, the plates were coated overnight with 10 μg/well DevS_201_ protein or left uncoated. The plates were then blocked overnight with BSA (5 mg/ml) followed by washing thrice with TBS (50 mM Tris–HCl, pH 7.5 and 150 mM NaCl). Thereafter, phage supernatants were added to coated wells and incubated for 1 hr. The plates were then washed vigorously with TBS containing 0.5% Tween 20. The bound phages were detected using HRP-conjugated anti-M13 antibody (Amersham Biosciences, UK) and o-phenylenediamine as a substrate and measurement of absorbance at A_490_. The extent of binding to DevS was calculated as A_490_ in DevS coated wells – A_490_ in control wells.

### DNA sequencing of phages

DNA of thirty high affinity binder phages was sequenced to determine the DevS binding peptide sequences. The desired peptides were then synthesized commercially with a purity level of > 98%.

### Activity of 'DevRS’ peptides in M. tb cultures

*M. tb* H37Rv was grown to logarithmic phase in Dubos broth containing 0.05% Tween 80 supplemented with 10% albumin dextrose complex (DTA medium) and sub-cultured with shaking at 220 rpm to A_595_ ~ 0.5. The culture was diluted in DTA medium (without Tween 80) to A_595_ ~ 0.025 for aerobic viability assays (REMA) and A_595_ ~ 0.005 for hypoxic assays (HyRRA) and used as described earlier [15].

### REMA

The REMA assay was performed in black, clear-bottomed, 96-well micro plates as described earlier [15]. Peptides were diluted in DMSO, and subsequent 2-fold serial dilutions were performed in 0.1 mL of Dubos medium supplemented with 0.05% glycerol in the micro plates. Approximately 5 × 10^4^ cfu *M. tb* bacteria were added per well. Appropriate control wells were included to calculate percentage inhibition of viability. The plates were incubated at 37°C for 64 h after which Resazurin (0.003% final concentration) and Tween-80 (1.25% final concentration) were added. The wells were observed after 24 and 48 h for a colour change from blue to pink and fluorescence of control wells >50,000 relative fluorescence units (RFU). Fluorescence was measured by excitation at 530 nm and emission at 590 nm using Gemini XS spectrofluorimeter in bottom reading mode. Percentage inhibition of viability was defined as: 1- (test well fluorescence/mean fluorescence of triplicate wells containing only bacteria) × 100.

### HyRRA

The HyRRA assay was performed as described earlier [15]. Briefly, 3 mL culture aliquots (A_595_ ~ 0.003 containing ~1.5 × 10^6^ cfu/mL *M. tb* H37Rv) were injected into 5 mL uncoated Vacutainer tubes and kept stationary at 37°C to allow for self-generation of hypoxia in the cultures. The peptides were injected into anoxic cultures (as judged by decolorization of methylene blue) at different concentrations on day 30 and the tubes were further incubated for 5 days at 37°C. Resazurin (0.0023% final concentration) and Tween 80 (0.66%/well final concentration) were injected into each tube and the tubes were incubated overnight. Culture aliquots (200 μl) were transferred to a 96-well black micro plate and fluorescence determined as described above.

### GFP Reporter assays

For the reporter assays, 200 μl *M. tb* culture aliquots harboring p3134c-1/pSigA reporter plasmids were used to measure GFP fluorescence in a spectrofluorimeter in bottom reading mode using an excitation wavelength of 483 nm and an emission wavelength of 515 nm as described [18].

### Auto phosphorylation of DevS_201_

DevS_201_ (15 μM final concentration) was incubated with 5 μCi of γ^32^P ATP (5000 Ci mmol^-1^, BRIT, India) in a 10 μl reaction mixture (containing 50 μM Tris HCl pH 8.0, 50 mM KCl, 10 mM MgCl_2,_ 50 μM ATP) at 25°C for 60 mins. The reactions were stopped by dilution with 200 μl of cold phosphate-buffered saline (PBS). The diluted contents were promptly transferred to a 96-well MultiScreen HA-plate prewetted with PBS and processed by high-throughput filtration using a vacuum manifold device to remove the reaction components and trap the proteins on the membrane [9]. The wells were washed thrice with 300 μl PBS using the vacuum manifold. The filters were subsequently dried on the vacuum for 2 mins at room temperature and individual filters were cut using the filter removal device. The radioactivity that was retained on each filter was quantitated using liquid scintillation counting. To monitor the effect of peptides on DevS_201_ autokinase activity, the peptides at various concentrations were preincubated with DevS_201_ in the reaction mixture for 30 mins at 25°C. ATP was then added to this reaction cocktail and tubes were incubated at 25°C for 1 h. The reaction was terminated by dilution with 200 μl of cold PBS and the samples were analyzed as described above.

### Phosphorylation of DevR/DevR_N_ by phosphotransfer from DevS_201_

To study the phosphotransfer reaction, DevS_201_ (15 μM final concentration) was phosphorylated for 60 mins as described above. Subsequently, full-length DevR or DevR_N_ protein (1–144 amino acid residues, 20 μM final concentration) was added to the phosphorylated DevS_201_ mix, and the reaction was incubated at 25°C for 2 mins. The reaction was terminated with 10 μl of 2X stop buffer (100 mM Tris-Cl, pH 6.8, 20% glycerol, 2% SDS, 280 mM β-mercaptoethanol, 0.01% bromophenol blue) and the samples were resolved on SDS-PAGE. The gel was rinsed in water and subjected to autoradiography.

### Cytotoxicity assay

The cytotoxicity of DevRS1 peptide was assessed in CHO (Chinese hamster ovary cells) and HepG2 (human liver hepatocellular carcinoma) cell lines. Both the cell lines were maintained in DMEM supplemented with 10% FBS at 37°C in 5% CO_2_. Approximately, 10^4^ cells were seeded per well in a 96-well plate and incubated at 37 °C for 12–16 h. The peptides were diluted in 125 μl DMEM and added onto cells (final volume 250 μl per well, 2.5 and 5 mM final peptide concentrations), and the plate was incubated at 37°C in 5% CO_2_ for 48 h. Subsequently, 20 μl of MTT (Sigma 5 μg μl^-1^) was added and incubated for 4–5 h at 37°C. Following incubation, media was discarded and the formazan crystals were solubilized by adding 200 μl DMSO and the absorbance measured at A_560_ nm. The percentage toxicity was calculated as:

$$1\;–\;\frac{A_{\text{peptide}}}{A_{\text{DMSO}}}\times 100$$

## Competing interests

The authors declare that they have no competing interests.

## Authors’ contribution

KK performed the biopanning procedure, phage screening and autokinase assays and drafted the manuscript. NKT carried out the reporter assays and REMA and HyRRA assays. SD participated in phage screening and performed the cytotoxicity experiments. JST contributed to the overall study design and manuscript preparation. All authors read and approved the final manuscript.

## Supplementary Material

Additional file 1 **'Pep’ peptides inhibit DevS autokinase activity.** To assess the effect of Pep A, B, C and D peptides on autokinase activity of DevS_201_, the peptides were incubated with DevS_201_ protein at 3 mM and 5 mM concentrations in an autokinase reaction. The reactions were analyzed in a high throughput format as described in Methods. The data represents the mean ± SD of three independent experiments. The number 0 on top of some of the bars indicates the lack of inhibition. The reproducibility was poor due to protein aggregation on addition of peptides to the autokinase reactions. Click here for file

Additional file 2 **Effect of DevRS A-ext and DevRS D on autokinase activity of DevS**_**201**_**.** The peptides were incubated with DevS_201_ protein at 5 mM concentration in an autokinase reaction and the reactions were then analyzed in a high throughput format as described in Methods. The data represents the mean ± SD of two independent experiments. Click here for file
