# Supplementary figures and images for: DevR (DosR) mimetic peptides impair transcriptional regulation and survival of Mycobacterium tuberculosis under hypoxia by inhibiting the autokinase activity of DevS sensor kinase
Source: BMC Microbiol. 2014 Jul 21;14:195. doi: 10.1186/1471-2180-14-195 (PMC4110071; doi:10.1186/1471-2180-14-195)

### Additional File 1

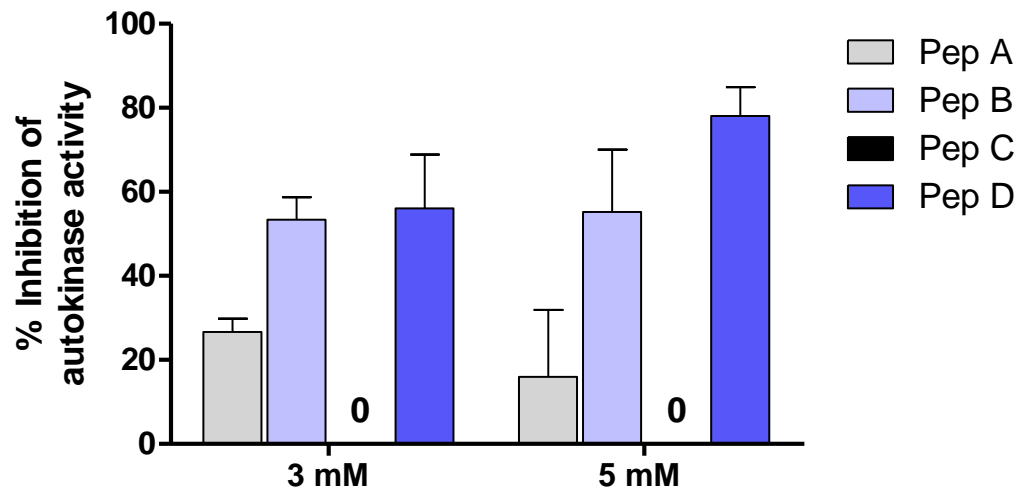

Supplement: Additional file 1 — 'Pep’ peptides inhibit DevS autokinase activity. To assess the effect of Pep A, B, C and D peptides on autokinase activity of DevS201, the peptides were incubated with DevS201 protein at 3 mM and 5 mM concentrations in an autokinase reaction. The reactions were analyzed in a high throughput format as described in Methods. The data represents the mean ± SD of three independent experiments. The number 0 on top of some of the bars indicates the lack of inhibition. The reproducibility was poor due to protein aggregation on addition of peptides to the autokinase reactions. [file 1471-2180-14-195-S1.pdf]

Additional file 2

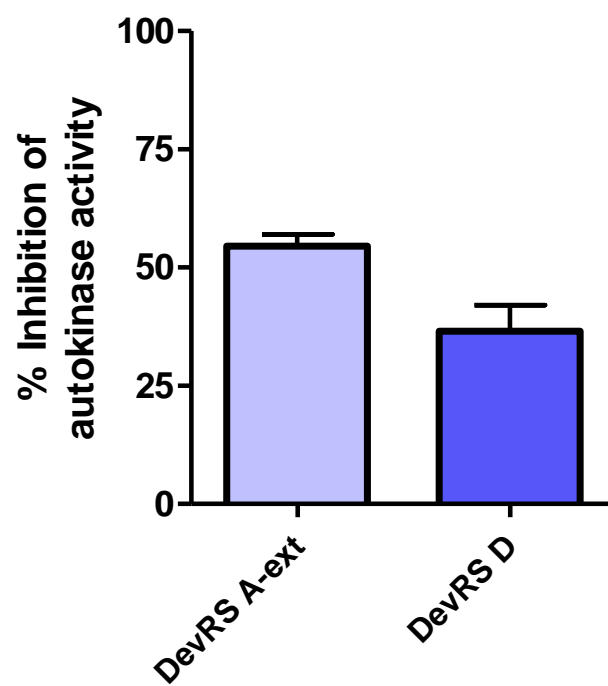

Supplement: Additional file 2 — Effect of DevRS A-ext and DevRS D on autokinase activity of DevS 201 . The peptides were incubated with DevS201 protein at 5 mM concentration in an autokinase reaction and the reactions were then analyzed in a high throughput format as described in Methods. The data represents the mean ± SD of two independent experiments. [file 1471-2180-14-195-S2.pdf]
